# Signal Transduction of Mineralocorticoid and Angiotensin II Receptors in the Central Control of Sodium Appetite: A Narrative Review

**DOI:** 10.3390/ijms222111735

**Published:** 2021-10-29

**Authors:** Michele Iovino, Tullio Messana, Giuseppe Lisco, Aldo Vanacore, Vito Angelo Giagulli, Edoardo Guastamacchia, Giovanni De Pergola, Vincenzo Triggiani

**Affiliations:** 1 Interdisciplinary Department of Medicine, Section of Internal Medicine, Geriatrics, Endocrinology and Rare Disease, University of Bari “Aldo Moro” School of Medicine, 70123 Bari, Italy; micheleiovino06@libero.it (M.I.); giuseppe.lisco@uniba.it (G.L.); aldo.vanacore4@gmail.com (A.V.); vitogiagulli58@gmail.com (V.A.G.); edoardo.guastamacchia@uniba.it (E.G.); 2 IRCCS, Istituto delle Scienze Neurologiche di Bologna, UOC of Pediatric Neuropsychiatry, 40139 Bologna, Italy; tullio.messana@isnb.it; 3 Unit of Geriatrics and Internal Medicine, National Institute of Gastroenterology “Saverio de Bellis”, Research Hospital, Castellana Grotte, 70124 Bari, Italy; giovanni.depergola@irccsdebellis.it

**Keywords:** sodium appetite, aldosterone, angiotensin II, organum vasculosum lamina terminalis, paraventricular nucleus, oxytocin, mesolimbic dopaminergic system

## Abstract

Sodium appetite is an innate behavior occurring in response to sodium depletion that induces homeostatic responses such as the secretion of the mineralocorticoid hormone aldosterone from the zona glomerulosa of the adrenal cortex and the stimulation of the peptide hormone angiotensin II (ANG II). The synergistic action of these hormones signals to the brain the sodium appetite that represents the increased palatability for salt intake. This narrative review summarizes the main data dealing with the role of mineralocorticoid and ANG II receptors in the central control of sodium appetite. Appropriate keywords and MeSH terms were identified and searched in PubMed. References to original articles and reviews were examined, selected, and discussed. Several brain areas control sodium appetite, including the nucleus of the solitary tract, which contains aldosterone-sensitive HSD2 neurons, and the organum vasculosum lamina terminalis (OVLT) that contains ANG II-sensitive neurons. Furthermore, sodium appetite is under the control of signaling proteins such as mitogen-activated protein kinase (MAPK) and inositol 1,4,5-thriphosphate (IP3). ANG II stimulates salt intake via MAPK, while combined ANG II and aldosterone action induce sodium intake via the IP3 signaling pathway. Finally, aldosterone and ANG II stimulate OVLT neurons and suppress oxytocin secretion inhibiting the neuronal activity of the paraventricular nucleus, thus disinhibiting the OVLT activity to aldosterone and ANG II stimulation.

## 1. Background

Thirst and sodium appetite represent a pivotal physiological function of all mammals, including humans. Drinking behavior regulates the salt-water balance and consequently renal metabolism, hormonal secretion, cardiovascular and cognitive functions [1]. In 1938, Curt Richter described sodium appetite in response to sodium deficiency by using animal models. More specifically, he discovered that adrenal gland ablation remarkably increased the salt intake requirement, and bilaterally adrenalectomized rats died five days after the sodium chloride infusion was withdrawn. This finding successfully demonstrated the relevance of the glomerulosa zone in the adrenal cortex in modulating salt intake through aldosterone secretion [2]. In addition, specific brain areas are involved in the regulation of water and sodium chloride intake, integrating signals arising from buccopharyngeal and gastric receptors via the trigeminal (V), facial (VII), glossopharyngeal (IX), and vagus (X) nerves. These afferences collect qualitative and quantitative gustatory water and sodium chloride detection, thus modulating motivated ingestion of both.

The perception of thirst and the need for salt intake occurs mainly at the anterior cingulate cortex (ACC) and insular cortex (IC), which receive signals from midline thalamic nuclei. Moreover, circumventricular organs (CVOs), such as organum vasculosum lamina terminalis (OVLT), subfornical organ (SFO), and area postrema (AP), lacking the blood-brain-barrier (BBB), detect changes occurring in blood osmolality and solute load. These mechanisms are responsible for appropriately maintaining extracellular fluid in a normal range and regulating synaptic inputs within the ACC/IC-medial thalamic nuclei network [3].

Fitzsimons [4] indicated two major thirst states: primary and secondary thirst. In turn, the primary thirst may be subdivided into the thirst of intracellular and extracellular origin because water is distributed both in the intracellular, typically for two-thirds, and extracellular, one-third, compartments. Sodium-ion plays a key role in regulating plasma osmolality and body fluids distribution, being the main cation of the extracellular compartment.

Intracellular thirst is induced by dehydration that, in turn, increases plasmatic osmolality. The “osmotic threshold” for thirst and vasopressin (VP) secretion is set to 275-295 mOsmol/kg [5,6]. Hyperosmolality is sensed by osmoreceptors in the CVOs, as OVLT, SFO and AP, and have efferent projections to VPergic neurons of the hypothalamic supraoptic (SON) and paraventricular nucleus (PVN). Since this connection, hyperosmolality stimulates the synthesis and release of VP transported via axoplasmic flow to the posterior pituitary (PP) [5]. VP released from PP acts on V2-renal receptors stimulating renal water reabsorption via translocation of aquaporin-2 vesicles from intracellular space to the plasma membrane of collecting duct epithelial cells [7]. In addition, thirst centers are also activated, inducing the individual to drink water. Therefore, the reabsorption of water and sodium in the kidneys and drinking water restore plasma osmolality.

Extracellular thirst is induced by hypovolemia. The “threshold” at which cardiovascular volume depletion stimulates drinking behavior and VP release is around 3% plasma volume reduction [8]. Peripheral signals from baroreceptors are located in the carotid sinus, aortic arch, left atria, and renal efferent arterioles. Signals are transmitted to cerebral thirst centers through the IX and X nerves, which project to, and synapse in, the nucleus of the solitary tract (NTS) of the dorsal medulla oblongata [5,9]. The NTS contains caudally noradrenergic [10,11] and rostrally adrenergic cells [12]. These brainstem catecholaminergic neurons are involved in the control of cardiovascular functions and water homeostasis. Baroreceptors are mechanoreceptors sensory neurons activated by blood vessel stretch via hyperpolarization-activated cyclic nucleotide (HCN) channels inducing cell excitability [13]. Hypovolemia and hypotension decrease the firing of stretch baroreceptors via the inhibition of mechano-transduction produced by HCN channels and the reciprocal mechanical communication between arterial wall and nerve terminal [10]. Under resting conditions with normal plasma volume and blood pressure, baroreceptors inhibit VP release by the NTS. Sved and coworkers [14] showed that bilateral electrolytic lesion of the NTS induced a significant increase of VP secretion and blood pressure, thus showing a tonic sympatho-inhibitory role played by baroreceptors synapsing with the NTS by IX and X nerves [9]. Furthermore, presynaptic α 2-adrenergic stimulation of the NTS attenuates VP release induced by depletion of cardiovascular volume [15]. Therefore, in hypovolemia or decreased central venous pressure, the reduced firing of atrial/aortic/carotid stretch baroreceptors induces an increase in VP release [15]. At the same time, hypovolemia and hypotension stimulate the release of renin, an aspartyl-protease synthesized at the juxtaglomerular apparatus of the kidney. In turn, renin is responsible for the cleavage of angiotensinogen, an α2-globulin secreted by the liver, to form the decapeptide angiotensin I. Angiotensin I is cleaved by the Angiotensin-Converting-Enzyme, a dipeptidyl carboxypeptidase located on the epithelial cells surface in the lung and renal microvasculature synthesize the octapeptide angiotensin II (ANG II) [16,17]. ANG II acts by stimulating tissue-specific G-Protein-Coupled Receptors (GPCRs) classified into two distinct categories: AT 1R and AT 2R [18,19] located in the brain, pituitary, adrenal gland, and kidney. Two subtypes of AT 1R [20,21,22] have been identified, namely the AT 1aR and AT 1bR. The AT 1aR is involved in regulating aldosterone and VP secretion, sodium and water retention, vasoconstriction, and stimulation of the sympatho-excitatory pathway. The AT 1bR is involved in the control of drinking behavior predominantly.

To better explain the role of angiotensin II in controlling water intake and sodium appetite, some experimental models have been discussed. For instance, the intracerebroventricular (ICV) infusion of ANG II increases water intake and sodium appetite, and this effect is abolished by the AT 1R antagonist losartan [23,24], but not AT 2R antagonism [25]. It has been reported that ANG II stimulates water intake and salt ingestion through two separate signaling pathways: (1) Protein Kinase C (PKC), a G protein-dependent pathway, and (2) Mitogen-Activated Protein Kinase (MAPK), a G protein-independent pathway. More specifically, the PCK pathway regulates water but not salt intake; conversely, the MAPK pathway controls salt but not water intake. The pretreatment with chelerythrine, a PKC inhibitor, suppressed ANG II-induced water intake, but it did not affect salt intake. In contrast, pretreatment with a MAPK inhibitor attenuated ANG II-induced salt intake but showed a weak effect on water intake [26]. Several mechanisms by which MAPK can mediate salt intake in the brain should be considered. First, ANG-MAPK signaling stimulates salt intake by modifying neuronal excitability in OVLT, SFO or forebrain integration sites, as NTS and BNT, via regulation of potassium channel subunit [27]. Moreover, MAPK stimulates salt appetite by upregulating SFO and OVLT AT1R expression [28]. MAPK is also involved in the rearrangement of the cytoskeleton by increasing actin filaments’ density. Dynamic regulation of actin polymerization induces some cellular processes such as migration, morphogenesis, axonal germination, and endocytosis. During dehydration, ANG II stimulates actin polymerization by modulating plasma membrane phosphoinositides that regulate actin cytoskeleton dynamics [29,30,31]. Therefore, this ultrastructural neural plasticity would explain the avidity of sodium appetite.

ANG II acts at the glomerulosa area in the adrenal cortex and promotes mineralocorticoid hormones release, including aldosterone. Crosstalk between ANG II and aldosterone signaling pathways in controlling salt and water balance is well documented. Aldosterone reduces sodium excretion within renal tubules by stimulating the expression of sodium-potassium ATPase pump at the level of tubular cells in both the distal and collecting ducts. Moreover, aldosterone facilitates the action of ANG II in hypothalamic neurons and at the level of certain forebrain areas designated to enhance sodium chloride intake. The “facilitating” action depends on aldosterone’s attitude to improve the affinity of ANG II for its receptor binding, primarily AT 1R, ACE gene expression, and ANG II signaling in vascular smooth muscle cells [16,32]. Aldosterone secretion is stimulated by prolonged sodium deprivation in an ANG II-independent manner [33,34]. Furthermore, a low-salt diet affects aldosterone synthase (AS) gene expression [35]. Thus, dietary salt deprivation is the most potent stimulus for aldosterone secretion.

**Figure 1:**
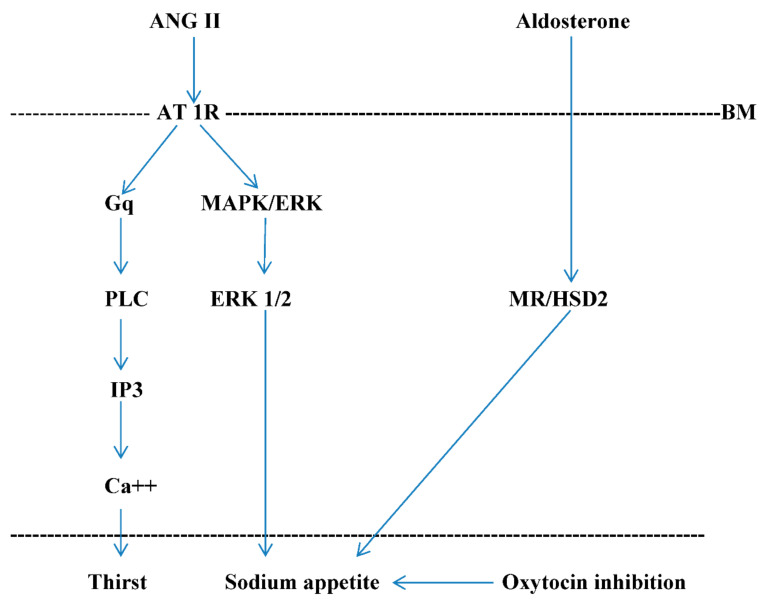
Cellular signaling pathways involved in sodium appetite. AT 1R signaling triggers the intracytoplasmatic production of the inositol 1,4,5-triphosphate (IP3) that enhances Ca++ mobilization and the extracellular regulated kinase 1 and 2 (ERK 1 and 2). Thus, IP3 stimulates thirst, while ERK 1 and 2 stimulates sodium appetite. Aldosterone induces sodium appetite, possess non-genomic and genomic effects on mineralcorticoid receptor (MR), and acts by the enzyme 11β-hydroxysteroid dehydrogenase type 2 (HSD2) that is co-localized with MR. Non-genomic MR effect induces a possible cytoplasmic crosstalk with ERK 1 and 2. Furthermore, aldosterone stimulates sodium appetite via inhibition of the responsiveness of PVN oxytocinergic neurons to ANG II. AT 1R: ANG II type 1 receptor—Gq: Gq protein α subunit—PLC: phospholipase C—MAPK: mitogen-activated protein kinase—BM: basal membrane of angiotensinergic and mineralcorticoid-sensitive cells.

In order to maintain the extracellular fluid (ECF) volume, aldosterone stimulates renal sodium reabsorption and sodium appetite by activating mineralocorticoid-responsive brain areas [36]. Centrally administered spironolactone, a mineralocorticoid receptor (MR) antagonist, minimizes sodium appetite and decreases sodium and water renal excretion in rats with heart failure [36]. The MR dissociates from chaperone proteins and undergoes nuclear translocation from the cytoplasm to the nucleus. Upon in the nucleus, MR interacts with some transcription factors, such as the activator protein-1 and nuclear factor-kB and regulates gene expression [37]. A central infusion of both aldosterone and ANG II leads to a synergistic increase in sodium appetite [38,39]. This synergy is selective for salt intake and independent of plasma sodium concentration. However, ANG II and aldosterone appear to have separate pathways to induce sodium appetite. Endogenous ANG II binds to AT 1R in the SFO and OVLT, which synapses with PVN and SON neurons [40,41,42]. AT 1R signaling triggers the intracytoplasmic production of the inositol 1,4,5-triphosphate (IP3) and enhances Ca^2+^ mobilization [43]. In addition, extracellular signal-regulated kinase 1 and 2 (ERK 1/2) is activated in the SFO and PVN after central administration of ANG II [44]. Thus, while the IP3 signaling pathway stimulates thirst by ANG II, the ERK 1/2 signaling is involved in sodium appetite [45]. Aldosterone acts by the enzyme 11β-hydroxysteroid dehydrogenase type 2 (HSD2) that is co-localized with the mineralocorticoid receptor (MR) in the NTS [46]. In order to better investigate the role of ERK 1/2 in ANG II- and DOCA-induced sodium appetite, the MEK inhibitor U0126 was demonstrated to reduced ANG II-induced salt intake significantly. However, it failed to reduce salt intake when a combined administration of ANG II and DOCA occurred. The Western blot analysis was used to measure the activation of ERK 1/2 phosphorylation in OVLT, SFO, PVN, and SON upon stimulation by ANG II and DOCA. ERK 1/2 phosphorylation was induced by ANG II but not DOCA in both OVLT-SFO and PVN-SON [47]. The role of IP3 was investigated using xestospongin-C (XC), an antagonist of the IP3 receptor. XC showed not to reduce salt intake induced by ANG II with DOCA reducing sodium ingestion similarly to that induced by ANG II alone [47]. Therefore, these data indicate that IP3 signaling plays an important role in the behavioral cooperativity of DOCA and ANG II to salt intake. Additionally, to investigate the neuronal activity of brain nuclei involved in sodium appetite cFos immunoreactivity was also used. XC pretreatment inhibited IP3-induced cFos immunoreactivity significantly in relevant hypothalamic nuclei and CVOs. It was noted that cFos levels were significantly increased in the OVLT and suppressed in the PVN after a combined administration of ANG II and DOCA, thus suggesting that DOCA stimulates salt intake via attenuation of the responsiveness of PVN OT-ergic neurons to ANG II [48] (Figure 1).

## 2. Fluid Balance

Body fluid balance depends on both intracellular and extracellular concentrations of water and sodium. Therefore, water ingestion, sodium intake, and excretion ensure proper solutes concentration and normal blood volume. Extracellular osmolality is controlled by water ingested and excreted, whereas ECF, including blood plasma, depends on the total sodium body content [49]. Water ingestion can restore ECF losses only when plasma sodium levels are normal-to-high, such as when water losses exceed sodium losses. On the other hand, when fluid losses also affect sodium concentration, as observed in the case of hemorrhages, vomiting or diarrhea, water ingestion alone cannot restore a normal ECF volume until an adequate supplementation of sodium chloride has been administered. The kidney plays an important role in regulating sodium homeostasis. An expansion of ECF volume increases blood pressure values, and the kidney is prone to excrete sodium excess [50,51]. In contrast, a reduction in ECF volume induces arterial hypotension, so enhancing the kidney to retain sodium chloride through mineralocorticoid hormones release. Nevertheless, renal compensative mechanisms may be limited, and sodium chloride supplementation becomes essential for restore ECF volume adequately.

In mammalians, sodium chloride appetite is a well-conserved behavior in response to a sodium deficiency [52]. Animal models demonstrated that the hedonic preference for carbohydrate-containing solutions decreases in sodium deficiency conditions [53]. Maternal episodes of sodium deficiency can also influence sodium chloride appetite during pregnancy [54,55,56]. Infants whose mothers reported frequent moderate-to-severe vomiting episodes in pregnancy had higher salt intake than those whose mothers had rare episodes or no vomiting. This finding suggests that maternal dehydration during pregnancy may enhance salt appetite in infants.

In experimental conditions, bilaterally adrenalectomized rats drunk more hypertonic saline solutions (NaCl 3%) compared to healthy strains, as the consequence of a conservative mechanism to chronic sodium depletion by urine output. To confirm the role of mineralocorticoid depletion in increasing the daily sodium requirement, sodium appetite in bilaterally adrenalectomized rats declined remarkably upon aldosterone was administered [57] but recurred immediately after aldosterone withdrawal [58]. Other mechanisms enhancing sodium appetite include a protracted dietary sodium restriction [59], peritoneal dialysis [60], colloid-induced hypovolemia [61], and furosemide [62].

Clinical data showed increased salt appetite in children with congenital adrenal hyperplasia, specifically those with the 21β-hydroxylase deficiency [63]. Patients with the Gitelman syndrome, due to a mutation in the Na-Cl cotransporter resulting in reduced function of the thiazide-sensitive sodium-chloride symporter in the distal convolute renal tubule, ingest large amounts of salt for maintaining an adequate ECF volume and normal blood pressure [64]. Similarly, patients with heart failure, liver failure, kidney failure, and salt-sensitive hypertension display an excessive dietary salt intake, but in these cases, a large amount of sodium chloride ingestion may induce a relevant ECF volume expansion. In this cluster of patients, mineralocorticoid receptor antagonists (e.g., spironolactone) decrease sodium appetite [36], contributing to beneficial effects [65]. A restricted sodium load leads to a reduced sodium body content and consequently decreases ECF volume. Hypovolemia stimulates thirst directly proportional to the ECF volume restriction [66], and sodium chloride appetite [67]. However, sodium chloride appetite is not induced by hyponatremia per se. Of known, bilaterally adrenalectomized rats display hyponatremia due to mineralocorticoid deficiency and show the same increase in sodium chloride requirement even if hyponatremia is avoided by water deprivation. This condition induces depletion of intravascular fluid volume but does not affect plasmatic sodium concentration, thus suggesting that hypovolemia stimulates sodium chloride appetite by pathways not involving the adrenal medulla, and hyponatremia is a condition unnecessary for salt intake [57,68]. In normal conditions, a short-term (1–2 days) dietary sodium deprivation is not able to induce sodium appetite because aldosterone release reduces renal sodium losses. On the other hand, a more prolonged dietary sodium deprivation stimulates sodium appetite in a way that sodium chloride intake exceeds significantly urinary sodium losses [57].

A direct relationship of sodium concentration between cerebrospinal fluid (CSF) and blood plasma has been reported [69]. ICV infusion of hypertonic saline solutions decreased salt intake in sodium-deficient and hypovolemic animals [70,71]. Mannitol or sucrose, osmotically active sodium-free molecules, stimulated salt intake both in sodium-deficient and non-deficient sheep when centrally administered even if these molecules induce a reduction of CSF sodium concentration [70]. This observation suggests that the activation of brain low-sodium receptors stimulates sodium chloride appetite. On the other hand, when the reduction of CSF sodium was induced by infusion of water or glucose, a cell-permeable solute, sodium intake did not increase. This different response suggests that brain low-sodium receptors are located inside the brain, while high-sodium receptors outside the brain within CVOs lack the BBB [72]. In order to evaluate the mechanism regulating brain sodium concentration and consequently salt intake, Watanabe et al. [73] analyzed the putative sodium channel Nav2/NaG. Nav2 gene-deficient mice showed cFos immunoreactivity markedly elevated in CVOs, as SFO and OVLT neurons, compared to wild-type mice exposed to water deprivation. This finding was associated with an abnormal intake of hypertonic sodium chloride solution during dehydration and sodium-deficient conditions, thus suggesting that Nav2 channel gene plays a key role in controlling brain sodium concentration and salt intake.

Sodium appetite may be induced by hypovolemic thirst, mainly via ANG II, but water intake precedes the onset of salt intake, and only when a large amount of water has been ingested, sodium appetite is triggered. This suggests that water producing osmotic dilution in the ECF removes a dominant inhibitory signal blocking sodium chloride appetite [74]. Stricker and Verbalis [75] showed an important role of oxytocin (OT) as an inhibitory hormone of sodium chloride appetite. ICV injection of OT inhibits salt intake induced by hypovolemic stimuli. Acute hyperosmolality stimulates OT and 11-deoxycorticosterone acetate (DOCA) release, while dietary sodium deprivation decreases OT secretion and stimulates ANG II-induced sodium intake. A naloxone-induced OT release suppresses salt appetite consequent to hypovolemia. This effect is prevented by administering OT receptor antagonist or experimental destruction of hypothalamic OT-ergic neurons in mice. In an experimental condition, hypovolemia was induced by polyethylene glycol, and OT gene knock-out mice ingested an amount of sodium chloride thrice than controls [47]. ANG II excites hypothalamic OT-ergic neurons; on the other hand, DOCA inhibits the responsiveness of OT-ergic neurons induced by ANG II in the hypothalamic PVN and SON. This suggests that mineralocorticoids suppress the OT-induced inhibitory stimulus on salt intake, supporting the hypothesis that reduction of OT-ergic tone within the PVN and SON may be the mechanism by which mineralocorticoids stimulate sodium appetite [46]. Additionally, the role of PVN, the major source of OT synthesis, in ANG II- induced sodium appetite was investigated by lidocaine, a local anesthetic that affects membrane conduction in neurons by blocking Na^+^ channel. In this experiment, lidocaine administered in PVN suppressed the OT release, and ANG II stimulated salt appetite. This effect was observed when lidocaine and ANG II (but not lidocaine alone) were centrally administered synergically. As an additive mechanism, lidocaine increased cFos immunoreactivity induced by ANG II in the OVLT and decreased it in the PVN [76]. Therefore, combined DOCA and ANG II treatment inhibits cFos expression in the PVN, reduces OT release, and increases cFos immunoreactivity in the OVLT, thus leading to a significant increase in sodium appetite. In fact, lidocaine inhibiting the neurosecretory activity in the SON allows for a robust salt intake via disinhibiting neural activity in the OVLT. These data indicate that OVLT may be the target of the inhibitory action of OT on sodium appetite. In fact, OT receptor mRNA is localized in the OVLT [77], and OT-ergic axonal projections arising from PVN and SON send inhibitory signals to OVLT [78], serving sodium appetite. In addition to the role played by OT, the inhibitory control of salt intake may be induced by signals originating from post-prandial gastric distension transmitted through the vagus nerve to the brain. The inhibitory signals reaching the brain modulate a neural network processing information related to homeostatic balance that includes OVLT, SFO, amygdala, PVN, lateral parabrachial nucleus (PBN), NTS, and AP [79]. Moreover, several neuropeptides, centrally injected, blunts sodium appetite, and include adrenomedullin [80], atrial natriuretic peptide [81], cholecystokinin [82], serotonin [83], and somatostatin [84].

The anteroventral area of the third ventricle (AV3V) contains two CVOs, the OVLT and SFO, that play a role in drinking behavior and VP release and have specialized neurons with osmoreceptor activity. ANG II activates OVLT, and SFO neurons significantly increase during hypovolemia and are involved in regulating water intake and ANG II-induced VP release [85,86,87]. Injuries involving only the OVLT or SFO induce a partial restriction of salt intake stimulated by prolonged dietary sodium deprivation [88,89,90]. Conversely, lesions involving both the OVLT and SFO usually lead to a more significant decrease in salt intake [80]. CVOs regulate sodium and water intake through the sodium channel (Na-x) expressed mainly in the SFO and OVLT. Na-x knock-out mice ingest more saline solutions compared to wild mice after acute dehydration. In addition, when Na-x cDNA was transfected into SFO neurons of knock-out mice, they recovered from salt-avoiding behavior under dehydration [91]. Therefore, the sodium-sensitive channel in the SFO is involved both in the stimulation of thirst and in suppressing salt intake when hypernatremia is induced [74,92]. SFO neurons exert an inhibitory control on neurons in the AV3V region; therefore, when SFO is damaged, its inhibitory role is suppressed. Rats with experimentally-induced SFO injury exhibit a restricted intake in fluid and saline solutions than controls during the first 2 h after ad libitum water access. Conversely, similar saline and water intake were observed over the following 20 h [89], thus suggesting that SFO injury plays a primary role in the acute saline and water intake restriction after dehydration. Furthermore, sodium stimulates GABA-ergic inputs within the CVOs, which may inhibit salt intake and VP release [93,94].

Distinct neural mechanisms in the SFO are involved in the inhibitory control of thirst and salt intake. Neurons involved in thirst are inhibited during sodium depletion through cholecystokinin-mediated activation of GABA-ergic neurons. Neurons involved in regulating salt intake are inhibited during dehydration by activating another population of GABA-ergic neurons sensitive to Na channel signals. This showed that SFO plays distinct control mechanisms in water and salt intake [95].

The area postrema (AP), a medullary CVO, is located in the transition region between the 4th ventricle and the central canal. Experimentally-induced AP lesions (APX) in rats lead to a considerable intake of hypertonic saline solution and impaired water retention competence in response to a hypertonic load. Moreover, APX rats failed to maintain sodium retention and showed a significant reduction of VP release in case of intracellular dehydration [96,97,98]. When the lesion involves the NTS, adjacent to the medio-caudal portion of the AP, the largest salt intake occurs [32], thus showing that AP neurons suppress the activity of NTS neurons involved in regulating sodium appetite. NTS neurons express MR and the enzyme HSD2; therefore, they are mineralocorticoid-sensitive and are activated in case of sodium deprivation. The mineralocorticoid DOCA, was found to increase the neuronal activity of HSD2 receptors as showed by c-Fos immunoreactivity within the NTS. DOCA-treated rats after access to hypertonic saline solutions (sodium chloride solution, 3%) drank significantly more water than vehicle-treated rats [98]. The presence of both the MR and the HSD2 on NTS neurons is essential because glucocorticoids may bind to MR, but HSD2 confers to aldosterone the specificity for the MR binding [99].

Aldosterone-sensitive HSD2 neurons within the NTS are selectively activated by protracted sodium depletion independently to thirst [100]. Hypertonic sodium solution administration increased water intake in rats, and this attitude was associated with a relevant c-Fos immunoreactivity in the medial NTS neurons but not in the HSD2 neurons. On the other hand, during polyethylene glycol-induced hypovolemia, sodium deprived for four consecutive days induced a significant increase in c-Fos immunoreactivity in HSD2 neurons [101]. Subsequently, when sodium-depleted rats had access to drink saline solutions, the HSD2 neurons decreased their activity, and neurons in the NTS and AP showed increased activity after salt intake [98,101]. HSD2 neurons are activated by sodium depletion after bilateral adrenalectomy, thus suggesting that these neurons are not only aldosterone-sensitive [98]. Recent data showed that activation of HSD2 neurons in the NTS stimulates the consumption of hypertonic sodium chloride solutions in mice, independently to thirst or hunger, and have distinct downstream targets activated by sodium depletion [102]. HSD2 neurons in the NTS are activated by sodium depletion and showed sate-dependent pacemaker-like activity inducing salt intake in synergy with ANG II signaling pathway in the so-called “synergy hypothesis”. Stimulation of sodium appetite by HSD2 neurons in NTS is mediated by efferent projections to the bed nucleus of the stria terminalis (BNST), which is the effector site for ANG II receptors of SFO neurons. Therefore, the network between ANG II neurons in the SFO and HSD2 neurons in the NTS regulates neural circuitries involved in the control of sodium appetite [103]. The afferent inputs to HSD2 neurons in the NTS arise from the AP [104] and exert an inhibitory control of sodium intake [105]. These neurons receive afferent projection also from the central nucleus of the amygdala and from the hypothalamic PVN [106]. Using anterograde and retrograde neural tracing techniques, HSD2 neurons innervate the ventrolateral bed nucleus of the stria terminalis (BNST), the pre-locus coeruleus (pre-LC), the external lateral PBN, the midbrain tegmental area, the hypothalamic PVN, the central nucleus of the amygdala and the peri-aqueductal gray matter [106]. Interoceptive HSD2 neurons in the NTS send signals to excitatory pre-LC neurons characterized by prodynorphin (PDYD) or proenkephalin B and the transcriptor factor Foxp2, and linked to salt intake in mice [107]. Optogenetic stimulation of PDYN neurons induces a robust sodium chloride intake in sated mice [107], and this behavior declines when the light is turned off [107,108]. Additionally, retrograde virus tracing showed that peripheral chemosensory signals suppress sodium appetite through GABA-ergic neurons in the BNST that are activated after salt intake [107]. These HSD2 neurons efferent pathways may be implicated in the brain circuitry serving salt intake.

## 3. Forebrain Sites Are Involved in the Integration of Signals Regulating Sodium Appetite

Two inputs can regulate salt intake: signals arising from sodium chloride need and signals for sodium chloride detection that are carried to the central nervous system (CNS) by the peripheral gustatory nerves (VII, IX, and X).

The bed nucleus of the stria terminalis (BST). Afferent projections arising from angiotensin-ergic neurons of the SFO and OVLT and A2 noradrenergic cell group of NTS synapse in the ventrolateral BST [10,109,110]. In addition, the BST receives afferent projections from the pontine PB nucleus [111,112] and pontine sites expressing HSD2 neurons, such as the pre-LC [113]. Efferent projections from the BST innervate the substantia innominata, lateral hypothalamic area (LHA), hypothalamic PVN, amygdala, and thalamic paraventricular nucleus [114]. Between these brain sites, the HLA plays an important role in regulating salt intake since injury confined to this area inhibits sodium appetite in bilaterally adrenalectomized rats [115]. BST neurons may regulate salt intake through an antagonistic mechanism on OT-ergic projections arising from hypothalamic PVN and SON. BST neurons are predominantly GABA-ergic and synapse with OT-ergic neurons in the PVN and SON. Therefore, after sodium depletion, the activation of HSD2 neurons stimulates GABA-ergic tone that, in turn, inhibits hypothalamic OT-ergic neurons that play inhibitory control of sodium appetite [73,74,115]. Thus, electrolytic lesions of the BST significantly reduce sodium appetite. Moreover, before and after experimentally-induced BST injury, rats drank equivalent amounts of water following ANG II or hypertonic saline administration subcutaneously, thus showing that BST plays a specific role in sodium but not water appetite [116,117]. The α2-adrenoceptor blocker yohimbine stimulated salt intake [117] and prevented VP inhibition induced by clonidine during hypovolemia [15]. The stimulation of salt intake by yohimbine is reduced following an experimental injury of the BST, thus showing that the NA-ergic forebrain circuitry, including A1 and A2 neurons of the BST and NTS, plays an integrative role in the control of sodium appetite.

Salt intake and sodium appetite satiation are also under the control of neural chemosensory mechanisms. As an example, dietary sodium restriction enhances salty taste, whereas oral sodium detection inhibits sodium appetite rapidly. Neurons in the pre-LC express the opioid polypeptide hormone prodynorphin involved in chemical signal transduction and cell communication. The activation of these prodynorphin-ergic neurons induces a significant increase in salt intake, while their inhibition selectively declines salt intake. Using a combination of in vivo optical recording and gastric infusion, Lee and coworkers [104] observed that sodium taste but not sodium ingestion was a condition necessary for the acute regulation of prodynorphin-ergic neurons of the pre-LC and for sodium appetite satiation. Furthermore, retrograde virus tracing indicated that BST neurons might modulate chemosensory signals. These inhibitory neurons are stimulated by salt intake and transmit inhibitory synaptic inputs to sodium-appetite neurons, thus suggesting that the interaction between prodynorphin-ergic and GABA-ergic neurons balances salt intake [104,105].

The Amygdala. The central nucleus of the amygdala innervates various parts of the BST [113] and regulates salt, water, and food intake. Bilateral electrolytic lesions of the amygdalar cellular groups induced both an increase in sodium chloride intake (1.5%) when the lesion was confined to the cortical nucleus, and a decrease in it when the lesion destroyed the lateral and medial nucleus [118]. Moreover, electrical stimulation of the cortical nucleus reduced salt intake, whereas the stimulation of the lateral nucleus increased it [119]. In addition, cell body damage within the medial region of the amygdala induced the suppression of corticosterone-potentiated and aldosterone-induced sodium appetite, thus suggesting that the medial region of the amygdala plays a key role in salt intake induced by adrenal hormones [117,120]. Gustatory and visceral-motory afferent projections arising from the NTS and AP synapse with the amygdala by pontine PB nucleus [108,121], and axons of aldosterone-sensitive HSD2 neurons in the NTS synapse in the central nucleus of the amygdala [122]. These connections between the NTS and amygdala are bidirectional because axons arising from amygdalar cell groups synapse in the NTS. The administration of an MR antagonist in the amygdala inhibited DOCA-induced salt intake [123], thus suggesting that mineralocorticoid-sensitive neurons of the amygdala, interacting with the NTS neurons, modulate sodium appetite. Salt intake modulated by amygdalar cell groups may be induced by direct axonal connections to forebrain nuclei regulating motor functions [124,125], such as the mesencephalic reticular formation, substantia nigra, ventral tegmental area, and trigeminal premotor neurons in the reticular formation of the medulla oblongata. These forebrain nuclei play a key role in eating and drinking behavior since synaptic signals are sent to oral motor nuclei of the V, VII, and IX cranial nerves, implicated in the central neural control of swallowing and drinking [126,127].

The septal area or septum pellucidum has been recognized as an important forebrain structure regulating thirst and salt-water balance. The lesion of the septal nuclei produces a sustained increase in diuresis and water intake in several species [128,129,130,131,132,133,134,135]. The medioventral septal (MVS) nuclei are mainly involved in this regulation, as showed by electrophysiological and behavioral data [135,136]. Whereas experimentally-induced injury involving the lateral septum [133,137] triggers a transitory increase in urine output and water intake, the electrolytic ablation of MVS nuclei results in long-lasting polyuria, polydipsia, and hypo-osmolality [133,134,135,138]. In the latter case, polyuria also persisted after water deprivation since VP release was significantly attenuated [138]. Neuroanatomical data indicate that hypothalamic SON and PVN neurons, where VP is synthesized, receive afferent projections from the MVS, and lesions confined in this area can induce degeneration of some nerve endings in the SON [139,140] and PVN [141], lastly affecting neuro-secretive activity. Particularly, morphological changes in magnocellular neurosecretory neurons have been described, including cytoplasmic and axonal accumulation of unreleased during dehydration [142]. Therefore, VP release normally occurring in response to dehydration is markedly reduced in lesioned animals [120,124,142], thus showing a role played by MVS nuclei in the axoplasmic transport rather than in the synthesis, as neurosecretory granules accumulated in SON and PVN magnocellular neurons. Furthermore, MVS nuclei contain a high concentration of ANG II receptors, classified into AT 1R and AT 2R and angiotensin-ergic nerve endings [86,143,144]. ANG II is a powerful stimulus to thirst and sodium appetite and exerts its dipsogenic action via AT 1R as aforementioned [22]. Rats with a lesion of the MVS nuclei showed a blunted response of VP release to ICV injection of ANG II, and hyperdipsia was not potentiated, thus suggesting that the dipsogenic effect induced by ANG II is at least in part mediated by the integrity of the MVS nuclei [144,145]. Recent data [133] showed that ablation of MVS nuclei increased daily water intake significantly so that the hyponatremia and plasma hypo-osmolality persisted for up to three months after the injury. However, hyperdipsia of lesioned animals was not potentiated by intravenous hypertonic saline [121] or ICV ANG II administration [133,145], or water deprivation [133], thus indicating that mechanisms controlling the thirst satiation are significantly reduced after lesion of the MVS nuclei. Septal area, containing a high density of ANG II and mineralocorticoids receptors, may be involved in the central effects of ANG II and aldosterone [146]. It has been reported that subcutaneous colloid administration induces hypovolemia with consequent ANG II, renin, and aldosterone release, marked enhancement of thirst, and sodium appetite in septal-lesioned animals. Hyperdipsia significantly increased when the hypovolemia was markedly pronounced, and sodium appetite increased following aldosterone stimulation, thus showing that activation of septal mineralocorticoids and ANG II receptors may contribute to stimulate sodium appetite [147]. Angiotensin-ergic activation of MVS nuclei stimulated thirst and sodium appetite. This stimulation was inhibited by administering AT 1R but not AT 2R antagonists into the SON, thus suggesting that AT 1R are involved in thirst and sodium appetite induced by MVS angiotensin-ergic activation in SON [148]. Aldosterone, centrally administered, stimulates salt intake and sympathetic tone without affecting water intake. Chronic aldosterone infusion into the 4th ventricle significantly increased the daily intake of sodium chloride (0.3 M). This effect was prevented by the MR antagonist RU28318, thus indicating that MRs were required for biological response. These findings suggest that aldosterone, binding to MRs in the central nervous system, activates brain circuits involved in the control of salt appetite [149].

Lateral hypothalamic area (LHA). Lateral hypothalamic lesions cause aphagia and adipsia. It should be noted that adipsia without aphagia was obtained. In rats, lasting adipsia was observed due to lesions located in the lateral and dorsal region of the hypothalamus between the ventromedial nucleus and the dorsal premammillary nucleus [150]. On the other hand, water intake can be triggered by electrical stimulation of the hypothalamic nuclei, whose destruction causes adipsia [151]. Synaptic inputs arising from HSD2 neurons in the BST and pre-LC reach the dorsolateral LHA [115]. Electrolytic lesions centering in the dorsolateral LHA suppressed salt intake in response to DOCA administration and acute hyponatremia [152]. Osmotic stimulation with 2.5% sodium chloride increased the mRNA expression levels encoding for corticotropin-releasing hormone (CRH) in the LHA [153]. ICV administration of CRH increases sodium appetite [154]. Hence, hyperosmolality induced by sodium chloride overexposure stimulates CRH synthesis and release that regulate neural circuitry serving salt-water balance through the PBN as a major target of CRH neurons in the LHA [155]. Moreover, water drinking and salt intake may be triggered by cholinergic stimulation by the muscarinic agonist carbachol injected into the LHA [156].

The role of mesolimbic neural activity and dopamine (DA) synthesis in ANG II- and Aldosterone-induced sodium appetite.

The mesolimbic DA-ergic system may be involved in behavioral responses, such as motivation and reward and salt intake following sodium depletion. DA-ergic neurons in the ventral tegmental area (VTA) have efferent projections to the nucleus accumbens septi and to ventral pallidum that play a role to induce goal-directed movement [157]. Rats experiencing sodium depletion showed significant alterations of neuronal plasticity with changes in dendritic morphology at the level of nucleus accumbens septi [158]. In addition, sodium depletion and aldosterone reduce DA transporter activity in the nucleus accumbens septi [159]. Combined treatment with ANG II and aldosterone increased the avidity for salt compared with either hormone alone [160]. The mesolimbic DA-ergic system induces goal-direct movement, such as salt ingestion after sodium depletion [157,158]. The administration of ANG II, but not DOCA, increased neural activation in the nucleus accumbens septi and VTA, as showed by cFos immunoreactivity. This activation was also observed following lidocaine injection into the PVN. On the other hand, increased levels of phosphorylated tyrosine hydroxylase, the rate-limiting enzyme for the DA synthesis, in the VTA are observed mainly after DOCA treatment, thus suggesting that sodium appetite is induced by the combination of ANG II-induced mesolimbic neural activity and DOCA-induced DA synthesis [161].

## 4. Discussion and Conclusions 

**Figure 2:**
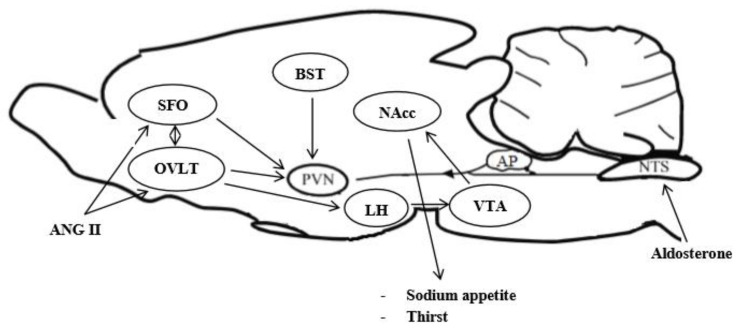
Midsaggital section of rat brain showing neural circuit for aldosterone and angiotensin II-induced sodium appetite. ANG II: angiotensin II—SFO: subfornical organ—OVLT: organum vasculosum lamina terminalis—PVN: paraventricular nucleus—AP: area postrema—NTS: nucleus of the solitary tract—LH: lateral hypothalamus—VTA: ventral tegmental area—NAcc: nucleus accumbens septi—BST: bed nucleus of the stria terminalis. BST express GABAergic inputs, VTA DAergic and PVN OTergic. ANG II acts via AT 1R in OVLT and SFO, while aldosterone via HSD2 neurons of NTS.

Most of the pharmacological studies herein reported having shown data that could be unreliable, considering that higher than normal concertation of ANG II, aldosterone, and OT have been centrally analyzed for the specific purpose to highlight the mechanisms of water and salt intake regulation (Figure 2). Therefore, possibly non-physiological results could have been obtained. Prolonged dietary sodium deprivation is a physiological non-invasive method to examine the mechanisms controlling sodium appetite. Sodium depletion stimulates salt intake significantly. Comparing the magnitude of ANG II and aldosterone-induced salt appetite with that observed following dietary sodium deprivation, the former is lower than the latter [162]. Dietary sodium restriction represents a more selective stimulus for salt appetite versus thirst induced by other pharmacological models using supra-physiological doses and stimulates the firing of HSD2 neurons that is inhibited after salt ingestion. Electrolytic lesion of the AP blunted sodium intake when the adjacent NTS is included in the lesion, thus showing that NTS neurons bordering the AP play an important role in regulating salt ingestion [163,164].

Inhibitory and excitatory pathways represent the neural circuitry involved in sodium appetite behavior. The NTS contains aldosterone-sensitive HSD2 neurons that activate specifically salt intake and synapse within the pre-LC, external lateral PB, and ventrolateral BST [165]. Inhibitory synaptic inputs arising from GABA-ergic neurons in the ventrolateral BST reach the hypothalamic PVN [166], and excitatory synaptic inputs from the PVN neurons projected to the NTS, PB, and central amygdala [167]. Therefore, it could be supposed that inhibitory axonal projections arising from the ventrolateral BST inhibit OT-ergic neurons during prolonged dietary sodium deprivation. Accordingly, sodium appetite could be induced by inhibition of OT-ergic tone in the PVN. However, ventrolateral BST neurons are also submitted to synaptic inputs arising from the SFO- and OVLT angiotensin-ergic neurons that play an additional stimulus to sodium appetite in response to sodium deficiency [108]. OVLT and SFO contain AT 1R that are activated by intravenous administration of ANG II, as shown by cFos- immunoreactivity [41]. Efferent axonal projections arising from the OVLT synapse in the LHA [168], where orexinergic neurons project to and synapse with DA-ergic neurons of the VTA, which project to the nucleus accumbens septi [169]. The administration of dopamine antagonists into the LHA suppresses salt intake. On the other hand, the stimulation of HLA induces DA release and consequently increases salt intake [170]. Mineralocorticoids acting on the NTS neurons may affect DA-ergic activity, thus stimulating sodium appetite. Therefore, combined ANG II plus aldosterone treatment increases mesolimbic neuronal and tyrosine hydroxylase activity, lastly leading to a highly motivated sodium appetite compared to water intake [103].

In conclusion, sodium appetite is under the control of signaling proteins, hormones, and brain areas. Signaling proteins involved in sodium intake are essentially MAPK and IP3. Pharmacological data showed that MAPK is specific for salt intake, as its inhibition suppresses sodium appetite without affecting thirst. Daniels et al. [23] showed that ANG II stimulates thirst and salt intake via MAPK pathway, whereas the combined treatment with aldosterone and ANG II induces sodium appetite via IP3 signaling. In addition, aldosterone might increase the responsiveness of OVLT neurons to ANG II by reducing the activation of MAPK signaling in some neurons and increasing IP3 in others. Aldosterone stimulates OVLT activation and suppresses the PVN neuronal activity, thus suggesting that OVLT plays an important role in enhancing sodium appetite by inhibiting OT release from PVN, disinhibiting the OVLT activity to aldosterone and ANG II stimulation. Histological evidence shows OT-ergic pathways from the PVN to the OVLT [77], and studies lesioning the OVLT induce different behavioral effects based on the size and localization of the lesion. For example, lesioning OT-ergic pathways from the PVN synapsing in the OVLT sodium appetite increases [78].

Additional pathways may be involved in aldosterone-related control of OT release from the PVN. Tracing studies showed the role played by the BNST in the control of synaptic inputs from the NTS to the PVN [171]. BNST GABAergic neurons receiving inputs from the NTS send inhibitory signals to PVN OT-ergic neurons [111].

Finally, the mesolimbic DA-ergic system represents a brain area for ANG II and aldosterone to augment the drive for sodium appetite playing a role as a modulator of selective motivation for sodium compared to water. Combined DOCA plus ANG II treatment induces cFos immunoreactivity in the VTA and nucleus accumbens septi and increases VTA tyrosine hydroxylase phosphorylation which induces the enzyme to synthesize more DA, provoking enhanced motivation for salt intake. Lidocaine in combination with ICV ANG II increases mesolimbic DAergic activation following its injection into the PVN, similar to that observed in OVLT [160].

Hence, the main points involved in the control of sodium appetite can be summarized as follows: (1) signaling proteins regulating aldosterone- and ANG II-induced sodium appetite such as MAPK and IP3; (2) brain areas and the neuronal circuitry connecting OVLT, PVN, BNST, with the mesolimbic DA-ergic system.

Furthermore, interoceptive neurons and their downstream neural circuits, such as pre-LC, show that taste signals play a major role in feed-forward signals from the peripheral. In depleted rats, post-ingestion sodium intake suppresses the activity of PDYN neurons (sodium appetite neurons). This mechanism is sodium-taste dependent since PDYN neurons activity is suppressed only by sodium intake. In addition, the antagonist of sodium channel amiloride inhibits the suppression of PDYN neurons activity induced by sodium intake as well as bypassing oral contact via intragastric sodium infusion PDYN neurons are not suppressed [172], thus suggesting that oral chemosensory signals modulate sodium appetite satiation via the facial nerves [106]. Neural circuits modulating sodium appetite are fast operating, thus inducing rapid appetite for salt intake. Moreover, sodium appetite neurons, PDYN neurons, received feed-forward signals from the periphery, thus suggesting that induction and satiation of salt intake play a role in the homeostatic balance of the body. In contrast, homeostatic imbalance may be involved in cardiovascular, renal, and hepatic diseases and obesity.
